# Mammalian-Specific Central Myelin Protein Opalin Is Redundant for Normal Myelination: Structural and Behavioral Assessments

**DOI:** 10.1371/journal.pone.0166732

**Published:** 2016-11-17

**Authors:** Fumio Yoshikawa, Yumi Sato, Koujiro Tohyama, Takumi Akagi, Tamio Furuse, Tetsushi Sadakata, Mika Tanaka, Yo Shinoda, Tsutomu Hashikawa, Shigeyoshi Itohara, Yoshitake Sano, M. Said Ghandour, Shigeharu Wakana, Teiichi Furuichi

**Affiliations:** 1 Laboratory for Molecular Neurogenesis, RIKEN Brain Science Institute, Wako, Saitama, 351–0198, Japan; 2 The Center for Electron Microscopy and Bio-Imaging Research and Department of Physiology, Iwate Medical University, Morioka, Iwate, 020–8505, Japan; 3 Support Unit for Neuromorphological Analysis, RIKEN Brain Science Institute, Wako, Saitama, 351–0198, Japan; 4 Technology and Development Team for Mouse Phenotype Analysis, RIKEN BioResource Center, Tsukuba, Ibaraki, 305–0074, Japan; 5 Advanced Scientific Research Leaders Development Unit, Gunma University, Maebashi, Gunma, 371–8511, Japan; 6 Laboratory for Behavioral Genetics, RIKEN Brain Science Institute, Wako, Saitama, 351–0198, Japan; 7 School of Pharmacy, Tokyo University of Pharmacy and Life Sciences, Hachioji, Tokyo, 192–0392, Japan; 8 Department of Applied Biological Science, Faculty of Science and Technology, Tokyo University of Science, Chiba, 278–8510, Japan; 9 Unite´ Mixte de Recherche 7357, Université de Strasbourg, Strasbourg, 67085, France and Department of Anatomy and Neurobiology, Virginia Commonwealth University, Richmond, Virginia, United States of America; Aix Marseille University, FRANCE

## Abstract

Opalin, a central nervous system-specific myelin protein phylogenetically unique to mammals, has been suggested to play a role in mammalian-specific myelin. To elucidate the role of Opalin in mammalian myelin, we disrupted the *Opalin* gene in mice and analyzed the impacts on myelination and behavior. *Opalin*-knockout (*Opalin*^−/−^) mice were born at a Mendelian ratio and had a normal body shape and weight. Interestingly, *Opalin*^−/−^ mice had no obvious abnormalities in major myelin protein compositions, expression of oligodendrocyte lineage markers, or domain organization of myelinated axons compared with WT mice (*Opalin*^*+/+*^) mice. Electron microscopic observation of the optic nerves did not reveal obvious differences between *Opalin*^*+/+*^ and *Opalin*^−/−^ mice in terms of fine structures of paranodal loops, transverse bands, and multi-lamellae of myelinated axons. Moreover, sensory reflex, circadian rhythm, and locomotor activity in the home cage, as well as depression-like behavior, in the *Opalin*^−/−^ mice were indistinguishable from the *Opalin*^*+/+*^ mice. Nevertheless, a subtle but significant impact on exploratory activity became apparent in *Opalin*^−/−^ mice exposed to a novel environment. These results suggest that Opalin is not critical for central nervous system myelination or basic sensory and motor activities under conventional breeding conditions, although it might be required for fine-tuning of exploratory behavior.

## Introduction

The advent of myelin-forming oligodendrocytes is phylogenetically crucial for vertebrates to acquire high-speed conduction velocity of nerve impulses in the central nervous system (CNS) [1]. Because mammals have larger brains with more complex neural connectivity than other vertebrates, one can expect that an evolutionary basis was acquired for the enhancement of mammalian-specific functions, such as the formation, maintenance, and plasticity of myelinated axons. *Opalin/Tmem10* encodes a CNS myelin protein [2–5] and has been identified as a phylogenetic-specific mammalian gene [6]. Therefore, we hypothesized that Opalin might play a mammalian-specific role in the oligodendrocytes and/or the formation of CNS myelin.

In myelinated axons of the CNS, the series of paranodal loops located in the outermost lateral region of the myelin sheath consists of a multi-lamellar membrane structure. The loops are non-compacted and are filled with a cytoplasmic channel that is continuous with the oligodendrocyte soma. Paranodal loops are located just beside the node of Ranvier and adhere to the axon via a continuous spiral of axoglial junction structures, called transverse bands. Adjoining paranodal loops are piled up with interposed extracellular space and have inter-membranous interactions via three types of autotypic junctional specializations: tight, gap, and adherens junctions [7–10]. These inter-membranous structures are thought to be involved in loop integrity, as well as communication or signaling between the loops. Opalin protein is located in the paranodal loops, as well as the oligodendrocyte soma and processes, suggesting a possible role for Opalin in the paranodal loop-loop interaction, but not the loop-axon interaction [3]. Opalin also undergoes a high degree of post-translational modification by *N*-linked and *O*-linked oligosaccharides, some of which are terminated with sialic acids [3]. Regional expression and the degree of sialylation in Opalin is age-dependent in the mouse brain, and may be associated with maturation, maintenance, and/or aging of the mammalian CNS myelin [11].

To elucidate the role of Opalin in myelinated axons of the CNS, we generated mice carrying a disruption of the *Opalin* gene (*Opalin*^−/−^ mice) in the present study. We assessed the role of Opalin in myelination and oligodendrocyte maturation and also evaluated their behavioral phenotypes. Surprisingly, the results showed that *Opalin*^−/−^ mice have no overt differences in myelination and oligodendrocyte maturation, as well as basic sensory and nociceptive functions and locomotion activity in the home cages. *Opalin*^−/−^ mice, however, show increased exploratory activity in novel environments. This study is an important step in elucidating the role of Opalin in mammalian-specific CNS myelin.

## Materials and Methods

### Animals

Two mouse lines, ICR and C57BL/6J, were purchased from Nihon SLC (Hamamatsu, Japan). All animal procedures were conducted according to the recommendations and protocols approved by the Animal Care and Use Committee of RIKEN (approval number: H21-2-244(4)) and Tokyo University of Science (approval number: N15006,7; N16008, 9). Animals were housed in an environment on a 12:12-hour light/dark cycle (daytime 8:00–20:00) with controlled temperature (23 ± 2°C) and humidity (55 ± 10%), as well as *ad libitum* access to food and water. Behavioral tests were performed in accordance with guidelines issued by the RIKEN Bioscience Technology Center in their “Outline for Conducting Animal Experiments.” All surgeries and dissections were performed under sodium pentobarbital or isoflurane anesthesia, and all efforts were made to minimize suffering.

### Generation of *Opalin* gene knockout mice (Opalin^tm1Tfr^)

**Fig 1:**
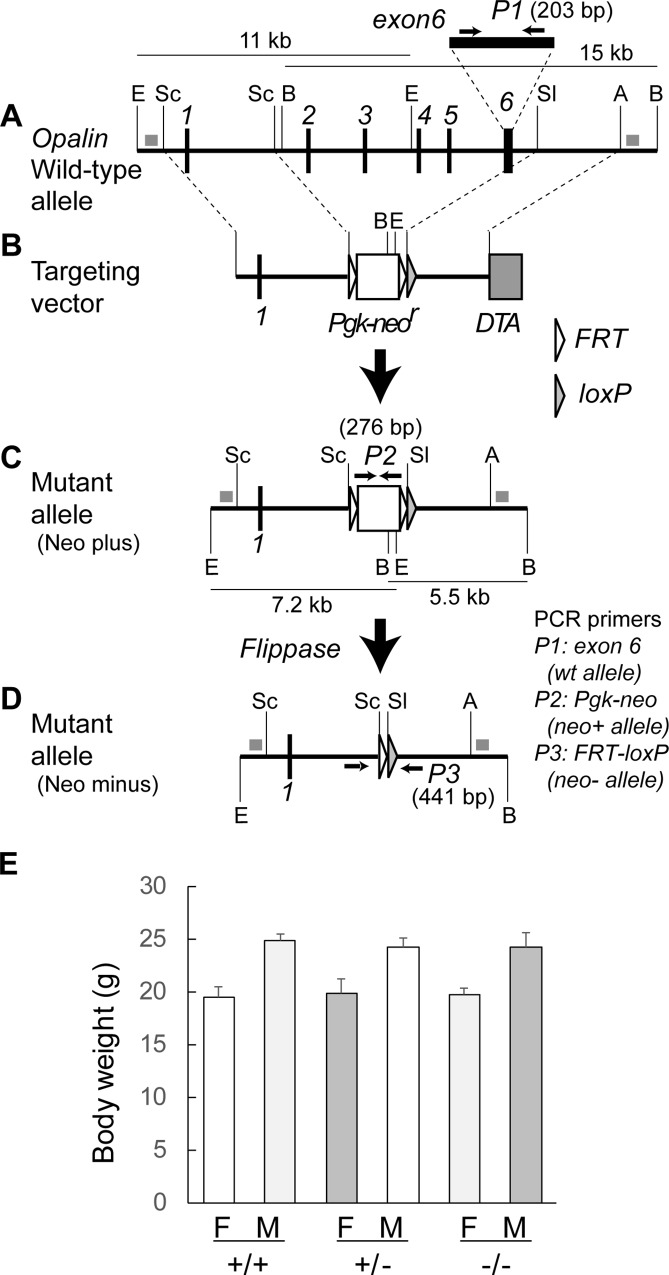
Generation and body weight of *Opalin*^*−/−*^ mice. (A) Genomic structure of mouse *Opalin* gene (WT allele). Six exons are indicated by thick vertical lines. To evaluate *Opalin* gene disruption, the exon 6 sequence of the WT allele was amplified by PCR using the internal primer set P1 (shown by arrows), which produced a 203-bp fragment present and absent in the WT and mutant allele, respectively. (B) Construction of targeting vector. A white box and a shaded box indicate the phosphoglycerate kinase 1 (*Pgk1*) gene promoter-driven neomycin resistance (*neo*^*r*^) gene (*Pgk-neo*^*r*^) and diphtheria toxin A (*DTA*) cassettes, respectively, flanked by the site-specific FLP recombinase recognition sites *FRT* (open arrow head). A *loxP* (shaded arrow head) sequence was present as a vestige of the construction in the right side of the *FRT-Pgk-neo-FRT* cassette and was not functional. (C) Structure of mutant allele (Neo plus). The region between exon 2 and 6 was deleted and replaced by the *FRT*-*Pgk-neo-FRT* cassette in the mutant allele. To detect the mutant allele, PCR using the primer set P2 produced a 276-bp fragment in the mutant, but not in the WT. (D) Structure of the *neo*^*r*^ cassette-deleted mutant allele (Neo minus). The *FRT-Pgk-neo-FRT* cassette was deleted by introducing the CAG promoter-driven flippase recombinase gene (CAG-FLP). PCR with the primer set P3 flanking the cassette produced a 441-bp fragment in the Neo minus line. In this study, the Neo plus mouse line was analyzed as a *Opalin* KO mice, because there were no overt changes between the Neo plus and minus lines. Thin vertical lines show restriction enzyme recognition sites: E, *Eco*RI; Sc, *Sca*I; B, *Bam*HI, Sl, *Sal*I; A, *Ava*I. (E) Body weight (g) of female (F) and male (M) *Opalin*^*+/+*^, *Opalin*^*+/−*^ and *Opalin*^−/−^ mice (8 wk). Seven animals for each sex and genotype were used. Error bars = SEM. WT = wildtype; KO = knockout.

A 21-kb genomic fragment containing exons 1–6 of the *Opalin* gene was isolated from a mouse 129/sv genomic DNA library cloned in bacterial artificial chromosome (BAC) vectors and used to construct the targeting vectors (Fig 1). For positive selection, the *Sac*I-*Sal*I fragment containing full-length *Opalin* exons 2–6 was replaced by the *Pgk1* (phosphoglycerate kinase 1) gene promoter-driven neomycin resistance gene (*Pgk-neo*) cassette [12], flanked by the yeast flippase (FLP) recombination target (*FRT*) sites (an unpaired and ineffective loxP fragment remained at the 3'-end of FRT due to construction strategy; see Fig 1). For negative selection, the diphtheria toxin A (DTA)-fragment gene cassette [13] was added to the 5′ end of the targeting vector. After transfection of 129/Ola-derived E14 embryonic stem (ES) cells [14] by electroporation, targeted clones were screened for G418 resistance and analyzed by Southern blot analysis. Chimeric mice were generated by injection of targeted ES cells into the C57BL/6J blastocysts. These methods (cloning of genomic DNAs from BAC library, construction of target vectors, Southern blot analysis of targeted ES cells, microinjection of ES cells into blastocysts) were performed as previously described [15, 16]. Male chimeras were mated with C57BL/6J females to obtain heterozygous mice. All engineered animals were backcrossed with C57BL/6J mice for more than six generations. To obtain the Neo minus line by deleting the *FRT-Pgk-neo-FRT* cassette, the 4.8-kb *Sal*I-*Bam*HI fragment (containing the CAG promoter-driven *FLP* recombinase gene) was prepared from the plasmid pCAGGS-FLPe and was microinjected into the pronucleus of fertilized eggs from the Neo plus line, as previously described [17]. In the present study, we analyzed the Neo plus line as Opalin KO mice, because there was no overt change between the Neo plus and minus lines in terms of morphological appearance and ordinary behavior.

### Mouse genotyping

Tail biopsy samples were obtained from mice at P10–14 and were digested in 100 μl proteinase K solution (0.5 mg/ml proteinase K, 0.1 mg/ml gelatin, 10 mM Tris-HCl, pH 8.3, 50 mM KCl, 2 mM MgCl_2_, 0.45% NP40, and 0.45% Tween20) overnight at 55°C. Following heat-inactivation at 100°C for 13 min, the digested samples were centrifuged at 17,360 × *g* for 3 min, and the resulting supernatant containing genomic DNA was analyzed by PCR. Genomic DNA solution (2 μl) was added to 18 μl PCR buffer containing forward and reverse primer sets (0.25 μM each), EX Taq polymerase (0.5 U/μl, Cat. RR001A, TAKARA, Kyoto, Japan), and dNTP mix (0.2 mM each) in EX Taq reaction buffer recommended by the manufacturer, and was amplified using a thermal cycler (GeneAMP PCR System 9700, Applied Biosystems, Foster City, CA, USA) with a sequential reaction: initial denature step at 94°C for 2 min, 33 cycles of the amplification step consisting of denature at 94°C for 30 s, annealing at 60°C for 30 s, and extension at 72°C for 30 s, with a final extension step at 72°C for 5 min. The forward (Fwd) and reverse (Rev) sequences of the primer sets P1 (to detect exon 6, WT allele), P2 (to detect neo plus, knockout allele), and P3 (to detect neo minus, Flpe-loxP) (Fig 1) were as follows: P1, Fwd: 5′-CAGCTGCCTCTCACTCAACA-3′ and Rev: 5′-CCAAAGGCAGACTTCTCTCG-3′ (product size = 203bp); P2, Fwd: 5′-ATGACTGGGCACAACAGACA-3′ and Rev: 5′-ATACTTTCTCGGCAGGAGCA-3′ (product size = 276bp); P3, Fwd: 5′-GGTGAGTGGGTTTTCTTGGA-3′ and Rev: 5′-CCAGGCTATGGAATGATGCT-3′ (product size = 441 bp).

### Antibodies

The rabbit anti-mouse Opalin antibody was generated in a previous study [3] (catalog no. RIK-B-OP, Cosmo Bio Co., LTD., Tokyo, Japan) and used at a concentration of 1 μg/ml. The following antibodies were also used: anti-pan voltage-dependent Na^+^ channel (Nav) (1:250) (mouse monoclonal K58/35, Cat. S8809 and rabbit polyclonal, Cat. S6936 from Sigma-Aldrich, St. Louis, MO, USA); anti-myelin-associated glycoprotein (MAG) (1:250) (mouse monoclonal, Cat. MAB1567 from Chemicon, Temecula, CA, USA, currently Millipore Corp., Temecula, CA, USA) for immunochemistry (IHC) and anti-MAG (1:500) (rabbit polyclonal from Santa Cruz Biotechnology, Inc., Santa Cruz, CA, USA) for Western blotting (WB); anti-PLP/DM20 antibody (1:1000) (rat monoclonal, clone AA3, a gift from Dr. K. Ikenaka); anti-myelin basic protein (MBP) (1:10000 for WB and 1:2000 for IHC, rabbit polyclonal, Cat. 16141, from IBL, Gunma, Japan); anti-Kv1.2 antibody (1:250) (mouse monoclonal, Cat. 05–408, from Upstate Biotechnology Inc., Lake Placid, NY, USA, currently Millipore Corp.); anti-NG2 (1:250) (rabbit polyclonal, Cat. AB5320, from Millipore Corp.); anti-Cx32 (1:50) (mouse monoclonal Cat. 35–8900, from Zymed, San Francisco, CA, USA, currently Invitrogen); anti-Claudin-11/OSP antibody (1:2000) (rabbit polyclonal, a kind gift from Dr. S. Tsukita [18, 19]); anti-Caspr antibody (1:250) (mouse monoclonal, a gift from Dr. E. Peles [20]); anti-Olig2 antibody (1/200) (rabbit polyclonal 1D-104, Cat. 18953 from IBL, Gumma, Japan); and anti-carbonic anhydrase II (CAII) antibody (1:500) (rabbit polyclonal [21, 22]). The secondary antibodies were as follows: horseradish peroxidase (HRP)-conjugated anti-rabbit IgG (H+L) (1:2000) (Cat. NA9340) and HRP-conjugated anti-mouse IgG (H+L) (1:2000) (Cat. NA9310) from GE Healthcare UK Ltd. The secondary antibodies for immunofluorescent staining were as follows: Alexa Fluor 488-conjugated anti-mouse IgG (Cat. A11029) (1:1000), Alexa Fluor 594-conjugated anti-mouse IgG (H+L) antibody (A11005), (1:1000) and Alexa Fluor 594-conjugated anti-rabbit IgG (H+L) (Cat. A11012) (1:1000) from Molecular Probes (Eugene, OR, USA). The secondary antibody used for diaminobenzidine (DAB) staining was biotinylated anti-rabbit IgG (1:1000) (Cat. BA-1000, from Vector Laboratories, Burlingame, CA, USA).

### Myelin fraction preparation

Myelin fractions were prepared as previously described [3, 23, 24]. Briefly, male mouse brains were homogenized in 14 volumes of Dounce homogenizer (w/v%) with ice-cold 0.32 M sucrose, 5 mM Tris-HCl (pH 7.4), and protease inhibitor cocktail (1× complete, EDTA-free; Roche Diagnostics GmbH, Germany). The homogenates (15 ml) were layered over 20 ml of 0.85 M sucrose and 5 mM Tris-HCl (pH 7.4) in a Beckman Coulter SW28 rotor and were centrifuged for 30 min at 75,000 × *g* at 4°C. The crude myelin layer at the interface of the two sucrose solutions was collected (~5 ml), resuspended in ice-cold deionized water to a final volume of 30 ml, and recentrifuged in a SW28 rotor at 75,000 × *g* for 15 min at 4°C. The pellet was resuspended and homogenized with Dounce homogenizer in 40 ml of ice-cold deionized water, and was centrifuged at 12,000 × g for 13 min at 4°C. The resultant pellet was again homogenized and recentrifuged at 12,000 × g as described above. The crude myelin membrane pellet was obtained and homogenized in 15 ml of ice-cold 0.32 M sucrose and 5 mM Tris-HCl (pH 7.4), layered over 20 ml of 0.85 M sucrose and 5 mM Tris-HCl (pH 7.4), and centrifuged in a SW28 rotor at 75,000 × *g* as described above. The myelin fraction at the interface between 0.35 M and 0.85 M sucrose was collected (about 5 ml), homogenized, and recentrifuged in a SW28 rotor as described above. The pure myelin membrane (pellet) was obtained and resuspended in 10 mM Tris-HCl (pH 7.4). The protein concentration was measured using a BCA protein assay kit (Pierce, Rockford, IL).

### Western blot analysis

Protein lysates were separated by SDS-polyacrylamide gel electrophoresis (SDS-PAGE) and electro-blotted onto nitrocellulose filter membranes (Hybond-ECL, Cat. RPN2020D, GE Healthcare UK Ltd., Buckinghamshire, England) as previously described [3, 24]. The blots were processed at room temperature by immersing for 1 h in blocking buffer consisting of 5% (w/v) skim milk (Snow Brand, Sapporo, Japan) in 1× PBS containing 0.1% (v/v) Tween 20 (PBS-T), followed by incubation in primary antibody in PBS-T containing 5% (w/v) skim milk for 1 h and, finally, incubation in HRP-conjugated secondary antibody for 1 h. After washing with PBS-T, the bound antibody was detected using the ECL Plus Western blotting detection reagent (Cat. RPN2106, GE Healthcare UK Ltd.) and X-ray film (MXJB Film, Cat. 864–8651, Eastman Kodak, Rochester, NY, USA).

### Immunohistochemistry

Immunohistochemical analysis was essentially performed as previously described [3]. Mice from both genders were anesthetized with diethyl ether and transcardially perfused with PBS, followed by 4% paraformaldehyde (PFA) in phosphate buffer (PB). The brains were resected, postfixed in 4% PFA at 4°C for 1 h, and cryoprotected by immersion in 20% sucrose in PBS overnight at 4°C. After embedding in Tissue-Tek OCT compound (Sakura Finetechnical, Tokyo, Japan), the brains were frozen in dry ice powder and cut into 10–16-μm sagittal sections at −20°C using a cryostat (CM1850; Leica Microsystems, Wetzlar, Germany). The sections were then air-dried for 1 h, rinsed three times in phosphate-buffered saline (PBS), and treated with methanol at −20°C for 20 min followed by three washes with PBS at room temperature for 10 min each. After blocking with 10% normal donkey serum (Cat. D9663, Sigma-Aldrich) in PBS containing 0.2% Triton X-100 (PBS-T), the sections were reacted with primary antibody in PBS-T containing 5% serum at 4°C overnight, then rinsed in PBS-T and incubated with Alexa Fluor- or biotin-conjugated secondary antibody in PBS-T at room temperature for 1 h, followed by PBS washes. The sections were then stained with the VECTASTAIN ABC kit (Vector Laboratories Inc., Burlingame, CA, USA) (0.5 mg/ml 3,3’-diaminobenzidine (DAB)/0.01% H_2_O_2_). Fluorescent-labeled and DAB-labeled sections were mounted using VECTASHIELD (Cat. H-1000, Vector Laboratories) and Permount mounting medium (Cat. SP15-100, Fisher Scientific, Pittsburgh, PA, USA), respectively. Immunoreactivity was examined using a fluorescent or light microscope (Eclipse E800; Nikon, Tokyo, Japan) equipped with a cooled CCD camera (SPOT; Diagnostic Instruments Inc., Sterling Heights, MI, USA), or a confocal laser microscope (LSM 510 META; Carl Zeiss, Oberkochen, Germany). Digital images were processed using Adobe Photoshop 6.0 software (Adobe Systems Inc., San Jose, CA, USA).

### Electron microscopy

The fine architecture of the myelin was analyzed using the rapid-freezing/freeze-substitution method to minimize artifacts caused by chemical fixed-ordinary electron microscopy. The *Opalin*^−/−^ mice and their WT littermate mice (8 weeks old) were deeply anesthetized with pentobarbital and decapitated. Small pieces of the optic nerve were quickly removed from the brain and frozen in liquid helium using a rapid freezing device (HIF-4K, Hitachi Instruments Service, Tokyo, Japan). Samples were freeze-substituted in 2% osmium tetroxide in acetone for 48 h at −80°C, followed by 2 h each at −20°C, 4°C, and room temperature. After washing in acetone, the samples were infiltrated with a mixture of acetone and EPON resin (TAAB, UK), passed through QY1 (*n*-butyl glycidyl ether, Nisshin EM, Japan), and embedded in pure resin. Ultrathin sections were cut on an ultramicrotome, stained with lead citrate and uranic acid, and examined by an electron microscope (H-7650, Hitachi, Japan or LEO 912AB, LEO, Germany).

### Behavioral tests

Male WT (WT, *n* = 7) and knockout (KO, *n* = 7) mice between 8 and 17 weeks of age were used for a series of behavioral tests performed using standard procedures from the Japan Mouse Clinic [25].

#### Hot plate test

A mouse was placed on a warmed plate to provide a nociceptive stimulus. Then, responses to heating were recorded. Latency to lick the hind paws was measured. The test was performed as previously described [26] with minor modifications. The hot plate test was performed using a commercially available apparatus consisting of a paraffin spreader (HI1220, Leica Camera AG) and acrylic cylinder (diameter, 11 cm; height, 25 cm). The plate temperature was adjusted to 52 ± 0.1°C before the test. We place a mouse into a cylinder on the hot plate, and latency from start of test to the initial licking of the hind paw was measured. The cut-off time was 60 s.

#### Tail flick test

The tail flick test was performed as previously described [26] with minor modifications using a commercially available apparatus consisting of a radiant-heat source and a photosensor for detection of the tail flick (model MK-330B, Muromachi Kikai Co., Tokyo, Japan). A mouse was restrained and the entire body was wrapped, except for the tail, in a sterilized cloth. The tail was then placed in a sensing groove. Immediately after, the temperature of the sensing groove was gradually increased by a radiant-heat source (beam intensity, 70). The latency, which was the median of three independent tests from the start of irradiation to the tail flick reaction, was measured. The cut-off time was 10 s to prevent damage to the tail.

#### Light/dark transition test

The light/dark chamber (O’Hara & Co., Ltd., Tokyo, Japan) consisted of a light chamber (200 mm long × 200 mm wide × 250 mm high) made of white vinyl chloride plates and a dark chamber of the same dimensions made of black vinyl chloride plates. The apparatus had an opening (50 mm wide × 30 mm high) in the middle of the wall that joined the two chambers. The opening was controlled by a guillotine door. Latency for entering into the lighted chamber and the number of transitions between light and dark chambers were measured [27]. The mice were tested at 8 weeks of age.

#### Open-field test

Each mouse (9 weeks old) was placed in the corner of an open-field apparatus (400 mm wide x 400 mm long x 300 mm high; O’Hara & Co., Ltd.) made of white polyvinyl chloride. The distance traveled by each animal in the open field was recorded for 20 min using a video-imaging system (Image OF9; O’Hara & Co., Ltd.) [27].

#### Home cage activity test

Each mouse (10 weeks old) was placed alone in a testing cage (227 mm wide × 329 mm long × 133 mm high) under a 12-h light-dark cycle (light on at 08:00 h) with free access to both food and water. After 1 day of acclimation, spontaneous activity in the cage was measured for 5 continuous days (starting at 08:00) using an infrared sensor (activity sensor, O’Hara & Co., Ltd.) [27].

#### Tail-suspension test

The tail of the mouse (14 weeks old) was fixed to a metal plate using adhesive tape. The mouse was then suspended from the metal plate in an experimental box (400 mm × 400 mm × 300 mm; O’Hara & Co., Ltd.). The duration of immobility was measured using a video-analysis system (O’Hara & Co., Ltd.) for 6 min.

### Statistical analyses

**Fig 2:**
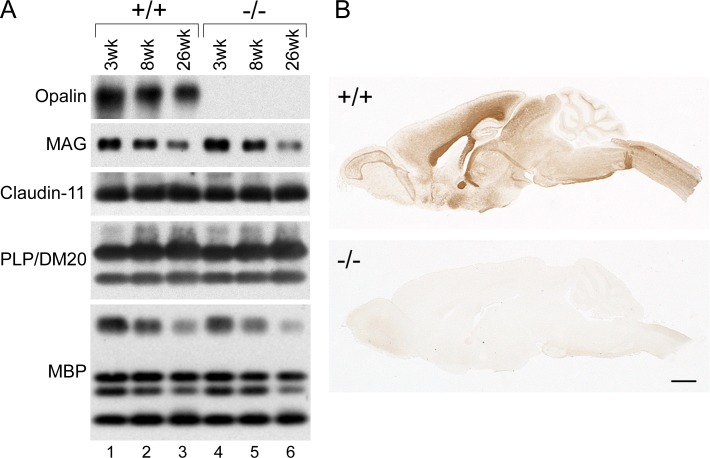
Contents of major myelin proteins are unchanged in *Opalin*^*-/-*^ mouse brains, except for Opalin loss, at weaning, young adult, and aged stages compared with WT mice. (A) Western blot analysis of myelin fractions from *Opalin*^*+/+*^ (lanes 1–3) and *Opalin*^−/−^ (lanes 4–6) mouse brains at 3 wk (weaning stage: lanes 1 and 4), 8 wk (young adult stage: lanes 2 and 5), and 26 wk (adult stage: lanes 3 and 6) using anti-Opalin, anti-MAG, anti-Claudin-11, anti-PLP/DM20, and anti-MBP antibodies. Opalin immunoreactivity is not detectable in *Opalin*^−/−^ samples as expected (top panel, lanes 4–6), although expression of the other tested myelin components is similar to the WT littermates. (B) Immunohistochemical analysis of *Opalin*^*+/+*^ and *Opalin*^−/−^ mouse brains at 16 wk using anti-Opalin antibody. *Opalin*^*+/+*^ mice show intense Opalin immunoreactivity. However, *Opalin*^−/−^ mice show no immunoreactivity for Opalin across various brain regions.

**Fig 3:**
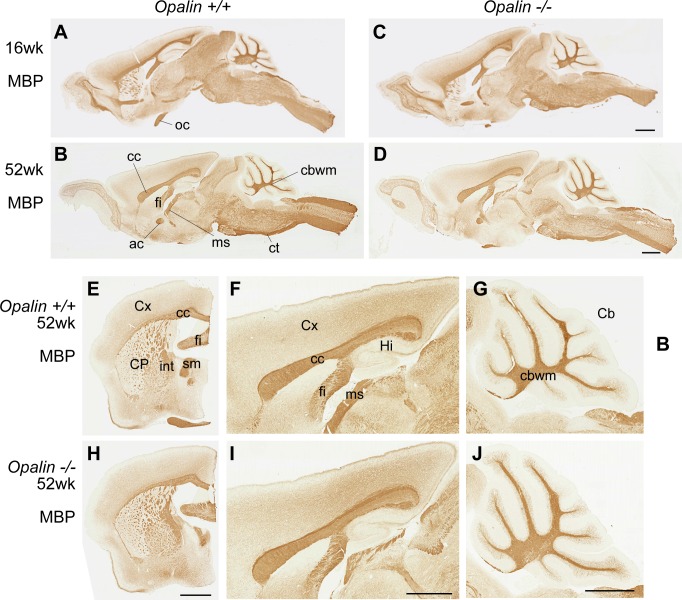
Distribution of myelin-rich regions is unaffected in *Opalin*^*−/−*^ mouse brains at young adult and aged stages compared with WT mice. Immunohistochemical analysis of *Opalin*^*+/+*^ (A, B, E–G) and *Opalin*^−/−^ (C, D, H–J) mouse brains at 16 wk (A, C) and 52 wk (B, D, E–J) using anti-MBP antibody. Immunostaining patterns for MBP at 16 wk and 52 wk do not show any differences between *Opalin*^*+/+*^ and *Opalin*^−/−^ mouse brains. Panels A–D, F, G, I, and J are sagittal sections. Panels E and H are coronal sections. Anterior commissure (*ac*), cerebellar cortex (*Cb*), cerebellar white matter (*cbwm*), corpus callosum (*cc*), caudate-putamen (*CP*), corticospinal tract (*ct*), cerebral cortex (*Cx*), hippocampal fimbria (*fi*), hippocampus (*Hi*), medullary atria of thalamus (*ms*), myelin basic protein (MBP), optic chiasm (*oc*), and wild type (WT). Scale bars, 1 mm.

**Fig 4:**
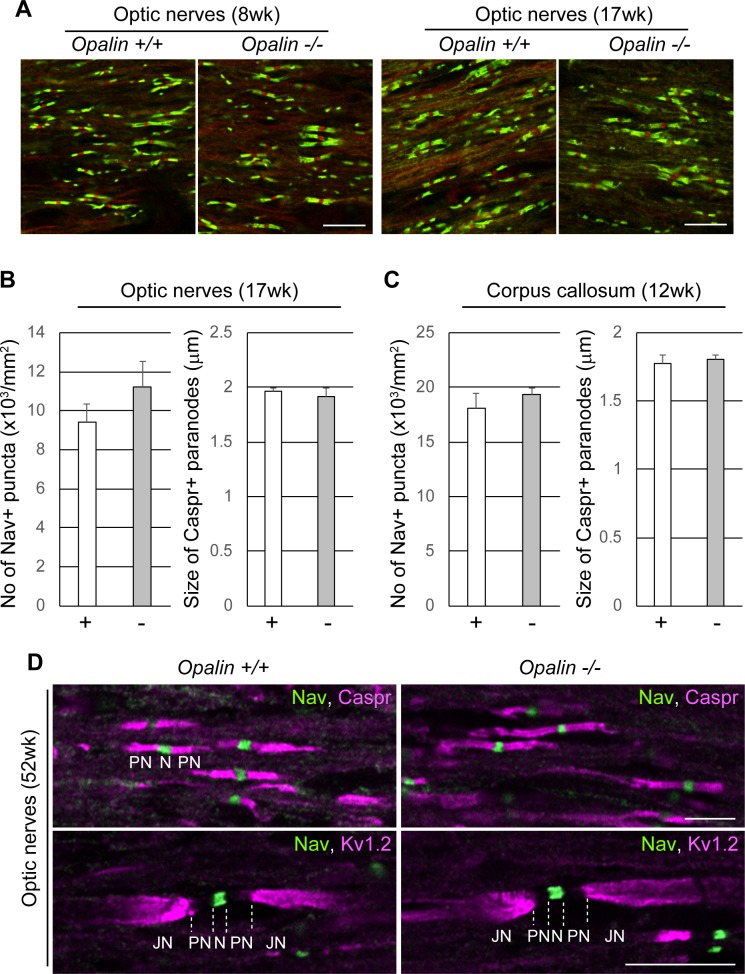
Polarized domain organization of myelinated nerves is not altered in *Opalin*^*-/-*^ mice compared with WT mice. (A) Optic nerves from *Opalin*^*+/+*^ and *Opalin*^−/−^ mice at 8 wk and 17 wk were immunostained with anti-Nav (red) and anti-Caspr (green) antibody. (B) Optic nerves of *Opalin*^*+/+*^ (+) (*n* = 3) and *Opalin*^−/−^ (−) (*n* = 3) mice at 17 wk were statistically analyzed. *Left*, number of Nav-positive puncta (Node of Ranvier) (per mm^2^). *Right*, size of Caspr-positive paranodes (longitudinal length of one side of the paranode) (in μm). Data are represented as mean ± SEM. (C) Corpus callosum of *Opalin*^*+/+*^ (+) (*n* = 5) and *Opalin*^−/−^ (−) (*n* = 4) mice at 12 wk were statistically analyzed. *Left*, number of Nav-positive puncta (per mm^2^). *Right*, size of Caspr-positive paranodes (in μm). Data are represented as mean ± SEM. (D) Myelin compartments in optic nerves from *Opalin*^*+/+*^ and *Opalin*^−/−^ mice at 52 wk were analyzed by co-immunostaining with anti-pan-Na^2+^ channel (Nav, marker for the Node of Ranvier [N]) and either anti-Caspr (marker for paranodes [PN]) or anti-K^+^ channel (Kv1.2, marker for juxtaparanodes [JN]) antibodies. Scale bars, 10 μm in both the top and bottom rows in A, 5 μm in the top row (for Nav and Caspr) in D, 10 μm in the bottom row (for Nav and Kv1.2) in D.

**Fig 5:**
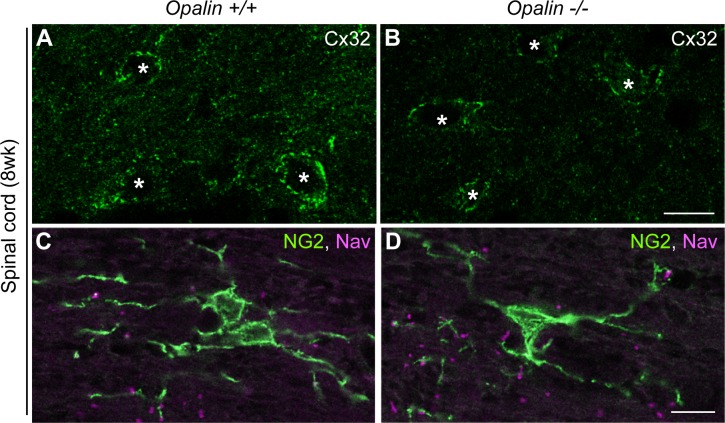
Immunostaining patterns for oligodendrocyte-astrocyte interactions and oligodendrocyte progenitor cells in spinal cords are unchanged in *Opalin*^*−/−*^ mice at 8 wk compared with WT mice. Immunostaining of spinal cords from *Opalin*^*+/+*^ and *Opalin*^−/−^ mice at 8 wk with anti-connexin 32 (Cx32, a component of gap junctions between oligodendrocytes and astrocytes and between paranodal loops) antibody (A and B) or with anti-Nav and anti-NG2 (marker for oligodendrocyte progenitor cells) antibodies (C and D). The immunostaining patterns of these markers shows no differences between *Opalin*^*+/+*^ and *Opalin*^−/−^ mice. Asterisks in A and B represent oligodendrocyte soma. Scale bars, 10 μm. WT = wild type.

**Fig 6:**
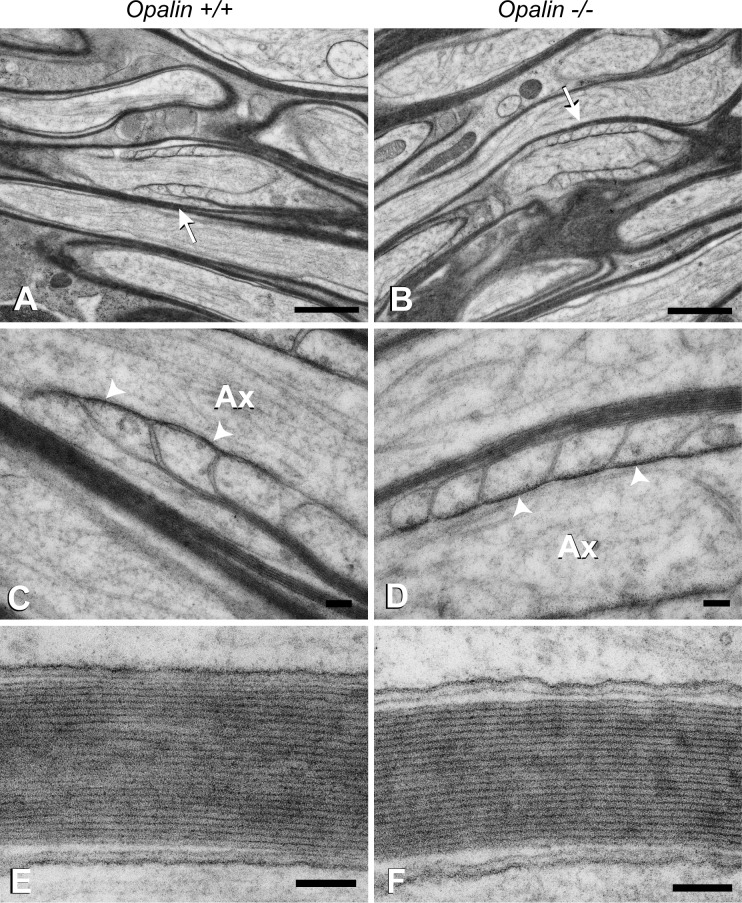
Electron photomicrographs of myelinated optic nerve axons of *Opalin*^*+/+*^ and *Opalin*^*−/−*^ mice. Each optic nerve axon from the *Opalin*^*+/+*^ and *Opalin*^−/−^ mice is firmly wrapped in a myelin sheath (A and B) with well-developed paranodal structures, such as loops, cytoplasmic swellings, and electron-dense transverse bands (*arrowheads*) juxtaposed to the axolemma (*arrows* in A and B, and enlarged in C and D). Periodical structures represented by the major dense and intraperiod lines of the myelin sheaths also appear to be similar between the *Opalin*^*+/+*^ and *Opalin*^−/−^ mice. Because the intervals between the paranodal loops subtly varied between samples, probably caused by slight differences in preparation, it was difficult to identify any change in this ultrastructure in the present study. Ax, axoplasm. Scale bars: 1 μm for A and B, and 0.1 μm for C through F.

**Fig 7:**
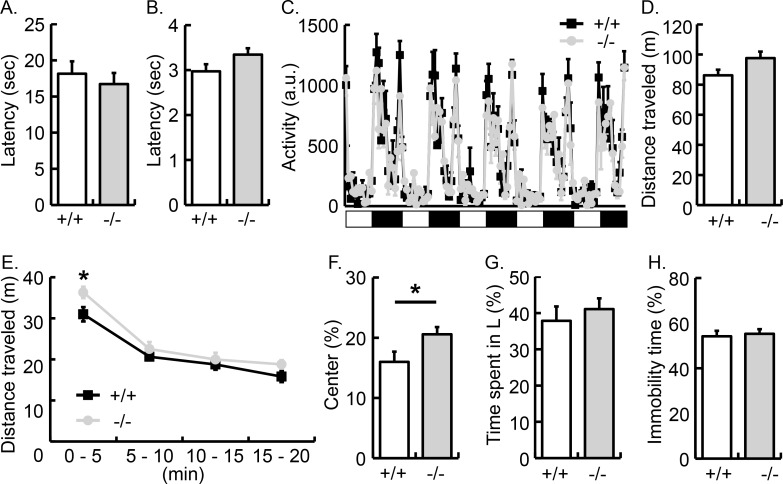
Behavioral phenotypes of *Opalin*^*-/-*^ mice. (A) Latency to lick the hind paws in the hot plate test (15 weeks old, *n* = 7/group). (B) Latency to flick the tail in response to heating (17 weeks old, *n* = 7/group). (C) Locomotor activity of *Opalin*^*+/+*^ (+/+; black square) and *Opalin*^−/−^ (−/−; gray circle) mice in home cage (10–11 weeks old, *n* = 7/group). Locomotor activities are shown by an arbitrary unit, one for each hour. White and black bars indicate day (light) and night (dark) periods, respectively. (D–F) Exploratory activity in a novel open field (9 weeks old, *n* = 7/group). (D) Total distance traveled for 20 min. (E) Locomotor activity each for 5 minutes. (F) Ratio of time spent in the center area. (G) Anxiety-like behavior in the light-dark box test (8 weeks old, *n* = 7/group). Ratio of time spent in the light arena. (H) Depression-like behavior in the tail-suspension test. Percentage of immobility time for 6 min (14 weeks old, *n* = 7/group). Data are represented as mean ± SEM. a.u., arbitrary unit; *p < 0.05. (*Opalin*^*+/+*^, white column; *Opalin*^−/−^ mice, gray column).

Data were analyzed using JMP software (SAS Institute, Cary, NC, USA), Excel (Microsoft, Redmond, WA, USA), or Statcel4 (OMS Publishing Inc., Saitama, Japan) software. All experiments in Figs 2–6 were performed at least three times for each condition. Error bars in Figs 1, 4 and 7 were produced using the standard error of mean (SEM) method. In Fig 1E, seven animals for each sex and genotype were analyzed. To compare the two genotypes in Figs 4 and 7, we used an unpaired two-tailed *t*-test. The data from a same animal were averaged and the number of mice was used as the number of samples in the analysis of Fig 4. We analyzed five or three different optic fields for each individual mouse in Fig 4B and four different optic fields for each individual mouse in Fig 4C. Differences were considered to be statistically significant when the probability value was < 0.05.

## Results

### *Opalin* gene disruption causes no abnormalities in gross anatomy or major myelin protein components

We generated *Opalin*-deficient mice (Opalin^tm1Tfr^) by deleting the genomic sequence containing exons 2–6 (Fig 1, and see Materials and Methods). Opalin homozygous (*Opalin*^−/−^) mice were born in the expected Mendelian ratio and grew normally. They did not exhibit any overt abnormalities in body shape and weight (Fig 1E), gross anatomy, visual inspection of ordinary locomotor activity and behavior (walking speed, hand grip strength, basic cognition [auditory, olfactory, visual, and tactile]), under standard breeding conditions (data not shown).

Western blot analyses of the myelin-related proteins showed that Opalin protein was completely absent from the myelin fraction of *Opalin*^−/−^ mouse brains at weaning (3 weeks of age [wk]), young adult (8 wk), and adult (26 wk) stages (Fig 2A). Conversely, there appeared to be no differences between *Opalin*^−/−^ mice and their WT (*Opalin*^*+/+*^) littermates in expression of myelin protein components, including myelin-associated glycoprotein (MAG) (a paranodal axon-glial interaction molecule), Claudin-11/OSP (a component of tight junctions in paranodal loops [18, 28]), proteolipid protein (PLP)/DM20 (a major component of myelin), and myelin basic protein (MBP) (Fig 2A). Quantitative analysis also revealed no significant change in MBP expression between *Opalin*^−/−^ and *Opalin*^*+/+*^ mice (S1 Fig).

Immunohistochemical analysis with anti-Opalin antibody revealed no Opalin protein expression in the brains of *Opalin*^−/−^ mice at 16 wk (Fig 2B). Additionally, the MBP immunostaining pattern across brain regions of the *Opalin*^−/−^ mice at 16 wk (Fig 3C) and 52 wk (Fig 3D, 3H–3J) showed no obvious difference compared with the *Opalin*^*+/+*^ mice at 16 wk (Fig 3A) and 52 wk (Fig 3B, 3E–3G), respectively. Together, these results indicated that deletion of the *Opalin* gene impaired expression of Opalin protein without altering expression levels of major myelin components throughout various brain regions.

### Loss of Opalin causes no alteration in the compartment of myelinated axons and oligodendrocyte maturation

We examined the effect of Opalin deficiency on nodal and paranodal domains through the use of double-immunostaining with anti-pan-Na^+^ channel antibody (Nav, a nodal domain marker) and anti-Caspr antibody (a paranodal domain marker) in the optic nerve (a central nerve) of *Opalin*^*+/+*^ and *Opalin*^−/−^ mice at 8 wk and 17 wk (Fig 4A). The overall appearance of Nav-positive nodal regions and Caspr-positive paranodal regions was not different between the genotypes in the optic nerve at the two different ages.

Statistical analyses indicated that the number of Nav-positive nodes and the longitudinal length of Caspr-positive paranodes in the optic nerves at 17 wk (Fig 4B) and the corpus callosum at 12 wk (Fig 4C) were also similar between both genotypes: number of Nav-positive puncta/mm^2^ in *Opalin*^*+/+*^ and *Opalin*^−/−^ (mean ± SEM) was 9,396 ± 965 (*n* = 3) and 11,241 ± 1,271 (*n* = 3), respectively, in the optic nerves (*p* = 0.31) and was 18,108 ± 1,295 (*n* = 5) and 19,335 ± 641 (*n* = 4), respectively, in the corpus callosum (*p* = 0.46); length of Caspr-positive signals (in μm) in *Opalin*^*+/+*^ and *Opalin*^−/−^ (mean ± SEM) was 1.97 ± 0.02 (*n* = 3) and 1.91 ± 0.09 (*n* = 3), respectively, in the optic nerves (*p* = 0.56) and was 1.77 ± 0.06 (*n* = 5) and 1.80 ± 0.04 (*n* = 4), respectively, in the corpus callosum (*p* = 0.70).

Opalin protein becomes hypersialylated with age [11], which may be related to the role of Opalin in the aged brain. Therefore, we also compared the optic nerves of the two genotypes at 52 weeks (Fig 4D). Dual immunolabeling with anti-Nav antibody and anti-Caspr or anti-K^+^ channel Kv1.2 (a juxtaparanodal domain marker) antibody showed that the node-paranode-juxtaparanode compartments were indistinguishable between *Opalin*^*+/+*^ and *Opalin*^−/−^ mice.

Endogenous Opalin is localized in the soma of oligodendrocytes and in the myelin loops [3]. We examined the cell-cell communication between oligodendrocytes and astrocytes by immunostaining for connexin 32 (Cx32), a component of gap junctions between oligodendrocyte soma and astrocytes [29, 30], in mouse spinal cords at 8 wk (Fig 5). Results showed no obvious difference in Cx32-positive puncta around the oligodendrocyte soma between *Opalin*^*+/+*^ (Fig 5A) and *Opalin*^−/−^ (Fig 5B) mice. We also examined oligodendrocyte progenitor cells (OPCs) in spinal cords at 8 wk by immunostaining with anti-NG2 antibody, confirming a similar distribution of NG2-positive OPCs between the spinal cords of *Opalin*^*-/-*^ mice (Fig 5D) and the *Opalin*^*+/+*^ mice (Fig 5C). Additionally, Olig2 and CA II oligodendrocyte lineage markers expressed in immature and mature oligodendrocyte were similarly distributed in the examined cerebellar white matter of 4-wk-old *Opalin*^−/−^ mice and *Opalin*^*+/+*^ mice (data not shown). Together, these results suggest no apparent alterations in oligodendrocyte and OPCs in young adult mouse brains (Fig 5).

### Loss of Opalin does not affect the fine architecture of compact myelin and paranodal loops

To examine the effects of Opalin deficiency on the fine structure of myelin, we evaluated optic nerves from 8-wk-old *Opalin*^*+/+*^ and *Opalin*^−/−^ mice using electron microscopy (Fig 6). Optic nerve axons in *Opalin*^−/−^ mice were firmly wrapped in a myelin sheath composed of well-developed paranodal structures, such as loops, cytoplasmic swellings, and electron-dense transverse bands juxtaposed to the axolemma (Fig 6B and 6D), which was similar to observations in the *Opalin*^*+/+*^ mice (Fig 6A and 6C). Moreover, an almost identical ultrastructure consisting of a periodicity of major dense and intraperiod lines of myelin sheaths was observed in *Opalin*^*+/+*^ (Fig 6E) and *Opalin*^−/−^ mice (Fig 6F). These results indicated that Opalin loss does not significantly affect the formation of basic paranodal and myelin structures.

#### Mice lacking Opalin show a subtle abnormality in exploratory behavior

To assess the effect of the Opalin loss in central myelin on mouse cognition and behavior, we implemented a comprehensive behavioral analysis. We first tested the response to sensory input in *Opalin*^−/−^ mice. Despite lacking a main component of myelin, the *Opalin*^*-/-*^ mice responded similarly to the *Opalin*^*+/+*^ mice in the hot plate and tail flick test, which test hyperthermia sensitivity in the hind paws and tail, respectively (Fig 7A, hot plate test, 15 wk, *n* = 7/group; *p* = 0.55; Fig 7B, tail flick test, 17 wk, *n* = 7/group; *p* = 0.11). Locomotion activities in the home cages (under 12-h light/12-h dark cycles) were similar over a 5-day period between both genotypes (Figs 7C, 10 and 11 wk, *n* = 7/group). Also, *Opalin*^−/−^ mice and *Opalin*^*+/+*^ behaved equally in the open-field test (Fig 7D; 9 weeks, *n* = 7/group; *p* = 0.07). These results suggested that *Opalin*^−/−^ mice had intact circadian rhythms and normal locomotor abilities. However, results from the open-field test showed that *Opalin*^−/−^ mice were more active in the first 5 min (Fig 7E; *p* < 0.05 in the 0–5 min bin) and spent more time in the center area compared with *Opalin*^*+/+*^ mice (Fig 7F; *p* < 0.05). Moreover, mice from both genotypes showed no significant differences in the light/dark transition (Fig 7G, 8 wk, *n* = 7/group; *p* = 0.56) and the tail-suspension test (Fig 7H, 14 wk, *n* = 7/group; *p* = 0.73). These results suggest that Opalin is not required for normal anxiety and depression-like behavior at least under conditions tested, but are involved in exploratory behavior in a novel environment.

## Discussion

Opalin has phylogenetically intriguing characteristics as a mammalian-specific CNS myelin membrane protein [2–6]. As such, we expected Opalin expression in oligodendrocytes and myelin to play an important role in the formation and/or function of the mammalian CNS. The present study investigated the functional role of Opalin by addressing whether Opalin deficiency in mice causes abnormal phenotypes, including myelin structures and behavior.

The present data showed that *Opalin*^−/−^ mice, maintained under conventional breeding conditions, do not exhibit impairments in expression of major myelin proteins, distribution of white matter regions across aged brains, polarized domain organization of myelinated axons, oligodendrocytes, and OPCs, and distribution and ultrastructure of myelinated axons. These results suggest that Opalin is not an essential component for oligodendrocyte maturation or for myelin formation. *Opalin*^−/−^ mice also showed no obvious abnormalities in the nodes or paranodes in the optic nerves, corpus callosum, cerebellar white matter (data not shown) or spinal cord (data not shown), consistent with normal basic visual, motor and nociceptive functions in behavioral tests. In addition, *Opalin*^−/−^ mice displayed normal levels of depression. Interestingly, however, *Opalin*^−/−^ mice showed an increased amount of time in the center of the open-field test, although there were no differences in light/dark transition properties compared with the WT mice. This suggests a subtle but significant impact on exploratory activity of *Opalin*^−/−^ mice when exposed to novel environments.

A previous study showed that although the gene locus of the human *Opalin* ortholog, *HTMP10*, is mapped on the chromosome 10q24 region responsible for temporal lobe epilepsy and spastic paraplegia, no disease-related mutations were found in the coding and splicing donor/acceptor sequences [31]. Our *Opalin*^−/−^ mice did not exhibit any clear epilepsy-like phenotypes or symptoms under ordinary breeding conditions throughout the present study, although we cannot rule out the possibility that the *Opalin*^−/−^ mice develop epilepsy under certain conditions. Recent studies showed decreased *Opalin* mRNA expression in the spinal cord of the progressive experimental autoimmune encephalomyelitis mouse model of multiple sclerosis [32] and in the frontal cortex of dogs with fucosidosis, a neurological lysosomal storage disease [33]. However, *Opalin* mRNA expression is upregulated in striatal tissues of mice carrying a mutation in LRRK2 (leucine-rich repeat kinase 2), a gene associated with familial and sporadic Parkinson’s disease [34]. Increased Opalin protein expression was also shown in a cuprizone-induced mouse model of demyelination in response to treatment with leukemia inhibitory factor [35]. Together with our preliminary data of chemically induced demyelination in male *Opalin*^−/−^ mice, we hypothesized that Opalin might be involved in demyelinating phenotypes.

It is noteworthy that although MAG-deficient mutant mice exhibit relatively subtle abnormalities, double mutants with mutated Fyn, a non-receptor-type tyrosine kinase downstream of MAG signaling, exhibit more severely abnormal phenotypes, suggesting the presence of compensatory mechanisms in the single mutants [36, 37]. Additionally, extensive back-crossing of a mixture of C57BL/6 and 129 inbred strains to generate the MAG null mutation in the C57BL/6 strain revealed clear evidence of CNS axonal degeneration in aging MAG-deficient mice [38], suggesting a genetic background effect on MAG-deficient phenotypes. Therefore, an Opalin knockout effect may become apparent by inbreeding mice to generate C57BL/6 *Opalin*^−/−^ mice or crossing to generate double mutants of *Opalin*^−/−^ and a related gene knockout. Moreover, we used only male mice for behavioral testing, to avoid an effect of the estrus cycle, but both genders for the other studies because gender-related differences in demyelination and remyelination [39] and behavioral phenotypes [40] have been reported. Sexual differences will be carefully examined in future studies.

## Conclusion

The knockout mouse from the present study demonstrates that the mammalian-specific and CNS myelin-specific membrane glycoprotein Opalin does not play a critical role in oligodendrocyte maturation and myelination, basic sensory and nociceptive functions, locomotor activity in the home cage, and anxiety- and depressive-like behavior. However, *Opalin*^−/−^ mice display increased exploratory activity in novel environments. Further studies are needed to clarify the potential biological significance of Opalin in myelin degeneration and regeneration, and more importantly in the fine-tuning mechanisms of learning and behavior.

## Supporting Information

S1 Fig Quantitative analysis of western blots suggests no significant change in MBP bands (1–4) between *Opalin*^−/−^ and *Opalin*^*+/+*^ mice at 3–8 weeks. Myelin proteins from three mice (N = 3) for both genotypes were analyzed by western blotting with anti-MBP antibody. Signal intensities of MBP bands were analyzed with ImageJ. Bars represent means ± SEM for bands 1–4, from top to bottom, in Fig 2A.
(TIF)
