# Supplementary figures and images for: Mammalian-Specific Central Myelin Protein Opalin Is Redundant for Normal Myelination: Structural and Behavioral Assessments
Source: PLoS One. 2016 Nov 17;11(11):e0166732. doi: 10.1371/journal.pone.0166732 (PMC5113975; doi:10.1371/journal.pone.0166732)

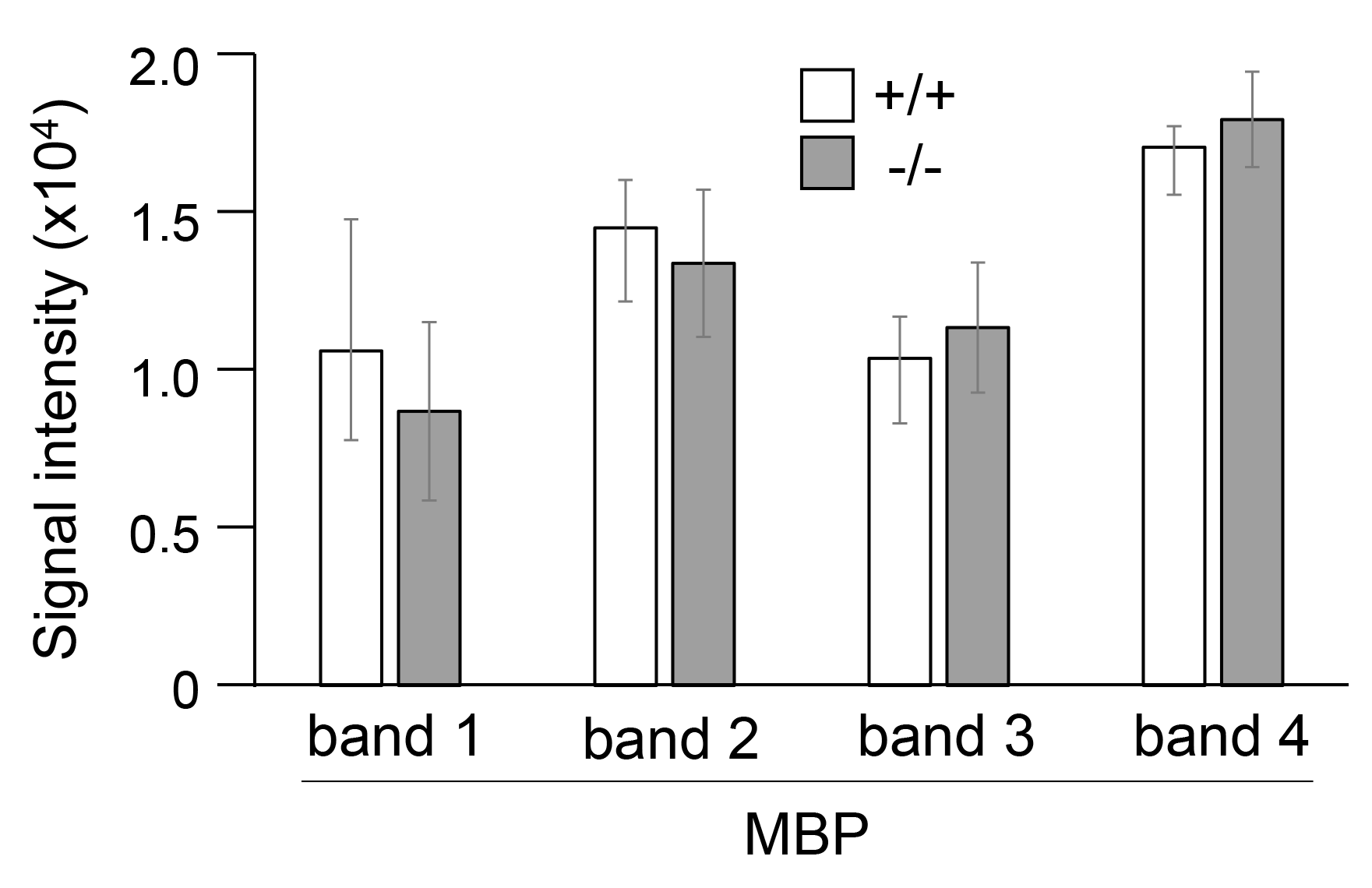

Supplement: S1 Fig — Myelin proteins from three mice (N = 3) for both genotypes were analyzed by western blotting with anti-MBP antibody. Signal intensities of MBP bands were analyzed with ImageJ. Bars represent means ± SEM for bands 1–4, from top to bottom, in Fig 2A. (TIF) [file pone.0166732.s001.tif]
